# Glycyl-alanyl-histidine protects PC12 cells against hydrogen peroxide toxicity

**DOI:** 10.1186/s12858-017-0089-x

**Published:** 2017-11-22

**Authors:** Hideki Shimura, Ryota Tanaka, Yoshiaki Shimada, Kazuo Yamashiro, Nobutaka Hattori, Takao Urabe

**Affiliations:** 1 Department of Neurology, Juntendo University Urayasu Hospital, 2-1-1 Tomioka, Urayasu, Chiba Japan; 2 Department of Neurology, Juntendo University School of Medicine, Tokyo, Japan; 3 Institute for Environment and Gender Specific Medicine, Juntendo University School of Medicine, Chiba, Japan

## Abstract

**Background:**

Peptides with cytoprotective functions, including antioxidants and anti-infectives, could be useful therapeutics. Carnosine, β-alanine-histidine, is a dipeptide with anti-oxidant properties. Tripeptides of Ala-His-Lys, Pro-His-His, or Tyr-His-Tyr are also of interest in this respect.

**Results:**

We synthesized several histidine-containing peptides including glycine or alanine, and tested their cytoprotective effects on hydrogen peroxide toxicity for PC12 cells. Of all these peptides (Gly-His-His, Ala-His-His, Ala-His-Ala, Ala-Ala-His, Ala-Gly-His, Gly-Ala-His (GAH), Ala-His-Gly, His-Ala-Gly, His-His-His, Gly-His-Ala, and Gly-Gly-His), GAH was found to have the strongest cytoprotective activity. GAH decreased lactate dehydrogenase (LDH) leakage, apoptosis, morphological changes, and nuclear membrane permeability changes against hydrogen peroxide toxicity in PC12 cells. The cytoprotective activity of GAH was superior to that of carnosine against hydrogen peroxide toxicity in PC12 cells. GAH also protected PC12 cells against damage caused by actinomycin D and staurosporine. Additionally, it was found that GAH also protected SH-SY5Y and Jurkat cells from damage caused by hydrogen peroxide, as assessed by LDH leakage.

**Conclusion:**

Thus, a novel tripeptide, GAH, has been identified as having broad cytoprotective effects against hydrogen peroxide-induced cell damage.

## Backgrounds

More than 7000 peptides have been identified as playing crucial roles in human physiology, including those acting as hormones, neurotransmitters, growth factors, ion channel ligands, or having anti- microbial activity [1–4]. Peptides are selective and efficacious signaling molecules that bind to specific cell surface receptors where they induce intracellular effects. Peptides represent an excellent starting point for the design of novel therapeutics. Even small peptides, such as dipeptides and tripeptides, may also have potent functions [5–8]. Some have cytoprotective functions and have been used in clinical trials for human disease [9]. Carnosine is a well-characterized antioxidant dipeptide composed of β-alanine and histidine. It has cytoprotective activity against various stresses as determined in both in vitro and in vivo models [10]. The imidazole ring of histidine is reported to have an important role in antioxidant cell protection [11]. Carnosine is a more effective singlet oxygen scavenger than L-histidine, although both compounds have been shown to protect against oxidative DNA damage and against liposome oxidation induced experimentally in vitro [12].

Histidine is a scavenger of hydroxyl radicals [13], and may interact chemically with toxic oxygen species through at least two distinct mechanisms: (1) by interfering with the redox reactions involving metal ions that produce the hydroxyl radical, and (2) by direct interactions of the histidine imidazole ring with singlet oxygen [14]. The imidazole ring of L-histidine has been shown to be responsible for the antioxidant activity of several biologically important dipeptides, including carnosine (β-alanyl-L-histidine), anserine (β-alanyl-3-methyl-L-histidine), and homocarnosine (l-aminobutyryl-L-histidine) [12]. Ala-His-Lys, Pro-His-His, and Tyr-His-Tyr were also reported to have antioxidant properties [15–17].

We hypothesized that histidine-containing tripeptides might also have antioxidant activity. In the present study, we synthesized and determined the antioxidant activities of tripeptides containing histidine and the small amino acids alanine and glycine.

## Methods

### Peptides

The histidine-containing tripeptides Gly-His-His (GHH), Ala-His-His (AHH), Ala-His-Ala (AHA), Ala-Ala-His (AAH), Ala-Gly-His (AGH), Gly-Ala-His (GAH), Ala-His-Gly (AHG), His-Ala-Gly (HAG), His-His-His (HHH), Gly-His-Ala (GHA), and Gly-Gly-His (GGH) were synthesized by and purchased from Biogate (Gifu, Japan). Carnosine was purchased from Sigma-Aldrich (St. Louis, MO, USA). GAH at 1 μg/μl = 3530 μM.

### Cell culture

PC12 (CRL-1721), SH-SY5Y (CRL-2266) and Jurkat cells (CRL-1990) were purchased from ATCC. PC12, Jurkat, and SH-SY5Y cells were grown at 37 °C (5% CO_2_ atmosphere) in RPMI 1640 medium supplemented with 10% heat-inactivated horse serum, 5% heat-inactivated fetal bovine serum, 2 mM L-glutamine, 1 mM sodium pyruvate, 100 U/mL of penicillin, and 100 mg/mL of streptomycin. Cell culture medium was changed three times per week, and when confluent, cells were split 1:6. For the experiments reported here, subconfluent cells were treated with different concentrations of 100–10,000 μM hydrogen peroxide, 10 μM staurosporine, or 500 μg/mL of actinomycin D.

### Lactate dehydrogenase assay

To assess cytotoxicity, lactate dehydrogenase (LDH) activity was measured using LDH cytotoxicity detection kits (Takara, Otsu, Shiga Japan). PC-12 cells were seeded into a 96-well plate at 2 × 10^6^ cells/mL with assay medium, for a period of 18 h at 37 °C in a 5% CO_2_ humidified incubator. The culture medium was then removed and replaced with serum-free medium and 1% bovine serum albumin (BSA) was added. The plates were treated with 100–5000 μM hydrogen peroxide for 1–24 h. After incubation, the samples were centrifuged for 10 min at 250 *g*. One hundred μL/well of supernatant was removed, without disturbing the cell pellet, and transferred into corresponding wells of a new 96-well plate. Solution C (100 μL, the reaction mixture) was added to each well and incubated for 30 min at room temperature. The 96-well plate was protected from light during this time. The absorbance of the samples was measured at 490 nm using the ARVO SX 1420 Multilabel Counter (PerkinElmer Wallac Inc., Turku, Finland).

### Flow cytometry analysis

Cells were washed twice with buffer (140 mM NaCl, 10 mM HEPES, 2.5 mM CaCI_2_, pH 7.4), resuspended in 1 mL of the same buffer, and incubated on ice for 30 min with 5 μL of propidium iodide (50 μg/mL H_2_O stock solution) added to each sample. They were then analyzed by flow cytometry (Becton Dickinson, San Jose, CA, USA) [18].

### Terminal deoxynucleotidyl transferase dUTP nick end labeling (TUNEL) assay

The TUNEL assay of PC12 cells was conducted using the Click-iT TUNEL Alexa Fluor 594 Imaging Assay Kit (Molecular Probes™, Eugene, OR, USA). DNase I was used to generate strand breaks in the DNA to provide a positive TUNEL reaction control. The number of Alexa Fluor 594-positive cells was counted using BZ-II Analyzer software (Keyence, Japan). Nuclear was stained with 4′, 6-diamidino-2-phenylindole (DAPI).

### Dead cell images

PC12 cells were incubated for 30 min at 37 °C with 4 μM of the EthD-1 (LIVE/DEAD viability/cytotoxicity kit reagent (Thermo Fisher Scientific Inc., Waltham, MA, USA). EthD-1 enters cells with damaged membranes and undergoes a 40-fold enhancement of fluorescence upon binding to nucleic acids, thereby producing a bright red fluorescence in dead cells (excitation/emission, ~495 nm/~635 nm, respectively). The number of Alexa Fluor 594-positive cells was counted using BZ-II Analyzer software (Keyence).

### Cell viability analysis

Cell growth was assessed using the Cell Counting Kit-8 (CCK-8; Dojindo, Japan) assay. The cells (2 × 10^4^) were plated in 100 μL of media and added to 100 μL of hydrogen peroxide with or without GAH or carnosine in each well of a 96-well flat-bottomed microtiter plate. Assays were done in triplicate cultures and incubated at 37 °C in an incubator with 5% CO_2_. Ten μL of the CCK8 solution was added to each well after 24 h of treatment, and the cells were cultured for another 2 h at 37 °C. The absorbance was measured using a microplate reader (Nanoquant Plate™; Tecan, Männedorf, Switzerland), at 450 nm with 600 nm used as the reference wavelength. The cell viability was expressed as a percentage of absorbance in cells with indicated treatments to that of the control cells.

### Statistical analysis

All values are expressed as mean ± SEM. One-way analysis of variance and post hoc Fisher’s protected least significant difference tests were used to determine the significance of differences between the groups. *P* values < 0.05 indicated significant difference.

## Results

**Fig. 1:**
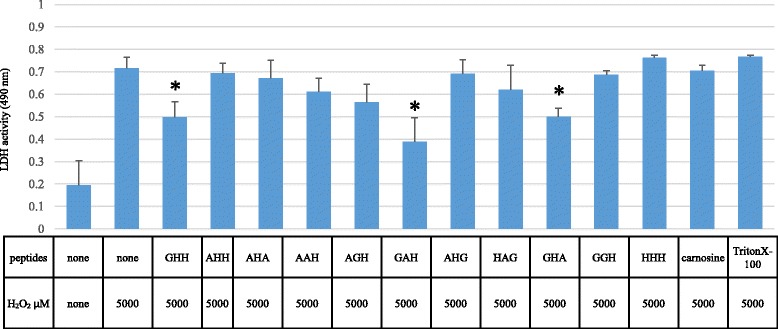
GAH protects against PC12 cell damage caused by hydrogen peroxide. The effect of histidine-containing tripeptides on hydrogen peroxide-induced cell damage as assessed by measuring lactate dehydrogenase (LDH) release into the medium in PC12 cells in culture. PC12 cells were incubated with 5000 μM hydrogen peroxide without or with 1 μg/μL of GHH, AHH, AHA, AAH, AGH, GAH, AHG, HAG, HHH, GHA, GGH, or AH carnosine for 4 h. PC12 cells were also incubated with Triton X100 as a positive control. The quantity of released LDH was estimated in the suspension aliquot from the 96-well plate. Measurements of the means ± standard deviation (S.D.) of at least four determinations for each sample were obtained by measuring at 490 nm. All of the above additives use the letter designation for the respective amino acid (i.e., H = histidine). * indicates significant difference (*p* < 0.001) between no peptide and carnosine

We incubated PC12 cell with 5000 μM hydrogen peroxide with 1 μg/μL of GHH, AHH, AHA, AAH, AGH, GAH, AHG, HAG, HHH, GHA, GGH, or carnosine for 4 h. GAH, GHH and GHA decreased LDH leakage compared with either no peptide or with carnosine (*p* < 0.01) (Fig. 1). GAH had the strongest hydrogen peroxide-induced cell death-inhibiting effect of all the tested peptides, and most effectively decreased cell damage in each experiment. We confirmed GAH cytoprotective activity in at least 20 independent experiments. We then analyzed the cytoprotective functions of GAH, compared to the other tripeptides used in this study.

**Fig. 2:**
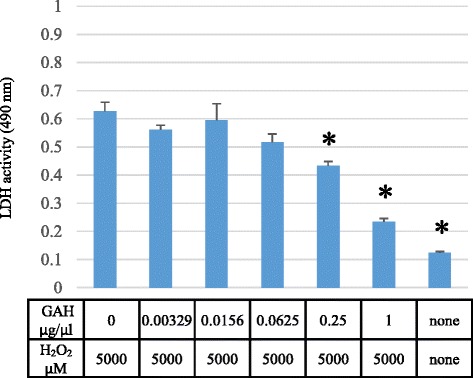
Cell protective effects activity of GAH are concentration-dependent. PC12 cells were incubated with 5000 μM hydrogen peroxide for 4 h without or with different concentrations of GAH (0.00329, 0.0156, 0.0625, 0.25, and 1 μg/μL). All data are shown as mean ± SE of three independent experiments. * indicates significant difference (*p* < 0.01) between no peptide. LDH = lactate dehydrogenase

**Fig. 3:**
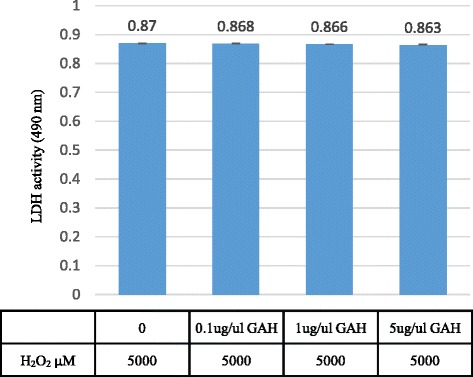
GAH does not directly inhibit LDH enzyme activity. We added 0.1 μg/μL, 1 μg/μL, or 5 μg/μL of GAH to the media of PC12 cells incubated with 5000 μM hydrogen peroxide for 4 h and subsequently measured the LDH activity. All data are shown as mean ± SE

To determine whether the cell protective activity of GAH was concentration-dependent, we incubated the cells with 5000 μM of hydrogen peroxide for 4 h without or with different concentrations of GAH (0.00329, 0.0156, 0.0625, 0.25, and 1 μg/μL). GAH at 0.25 μg/μL decreased the amount of LDH release (*p* < 0.01), and 1 μg/μL GAH was even more effective in this assay (*p* < 0.01) (Fig. 2). Thus, the highest concentration of GAH tested here had the best cytoprotective activity. We then determined whether GAH directly inhibited LDH enzyme activity. PC12 cells were incubated with 5000 μM hydrogen peroxide and culture media was collected and assayed. Different concentrations of GAH were added to the media and the LDH activity was determined. GAH at 0.1 μg/μL, 1 μg/μL, and 5 μg/μL did not inhibit enzyme activity (Fig. 3). These results indicate that GAH did not directly block LDH activity, but most likely prevented its leakage into the media, caused by loss of membrane integrity.

**Fig. 4:**
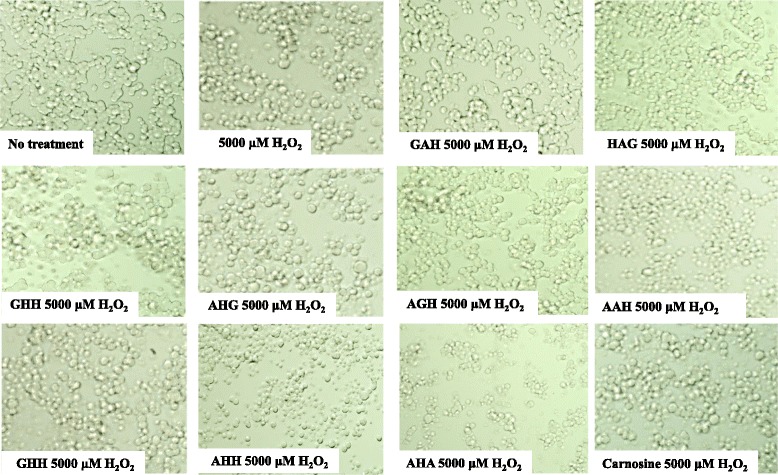
Micrographs representative of shape changes of PC12 cells. PC12 cells were incubated with 5000 μM hydrogen peroxide for 4 h with or without 1 μg/μL of GHH, AHH, AHA, AAH, AGH, GAH, AHG, HAG, HHH, GHA, GGH, or AH carnosine for 4 h

**Fig. 5:**
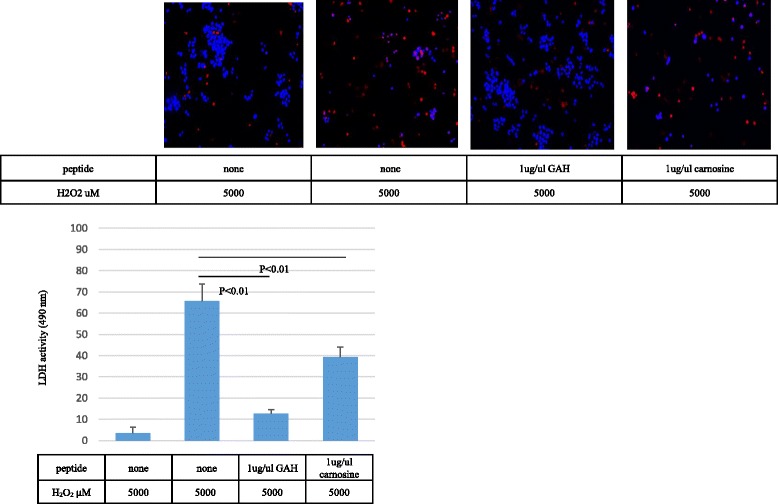
GAH reduces PC12 cell death as analyzed using ethidium homodimer 1. PC12 cells were incubated with ethidium homodimer 1 after treatment with 5000 μM of hydrogen peroxide for 4 h. Images in the upper panel are ethidium homodimer 1 staining (*red*) and the *lower* panel DAPI stained nuclei (*blue*) of PC12 cells. The number of dead cells per 100 cells is shown. GAH more effectively prevented cell death relative to no peptides and carnosine. All data are shown as mean ± SE of three independent experiments (*p* < 0.01). DAPI = 4′,6-diamidino-2-phenylindole

Cell morphology assessments showed that hydrogen peroxide treatment decreased the number of adherent PC12 cells and increased the number of nonadherent cells, resulting in a change from a flat-shaped to a round-shaped cell. The addition of GAH together with hydrogen peroxide increased the number of adherent cells and maintained the flat shape of the cell compared to the effect of hydrogen peroxide alone or after coculture with HAG, GHH, AHG, AGH, AAH, GHH, AHH, AHA, or carnosine (Fig. 4). We next examined the mechanism of cell protection by GAH using the ethidium bromide homodimer 1 from the LIVE⁄DEAD® Viability⁄Cytotoxicity Kit (Molecular Probes™^)^ to stain dead cells. PC12 cells were incubated with ethidium homodimer 1 after treatment with 5000 μM hydrogen peroxide for 4 h in the presence or absence of GAH. The number of dead cells per 100 cells was calculated. GAH and carnosine significantly protected against hydrogen peroxide-induced cell death (*p* < 0.01), GAH more strongly than carnosine (*p* < 0.01) (Fig. 5).

**Fig. 6:**
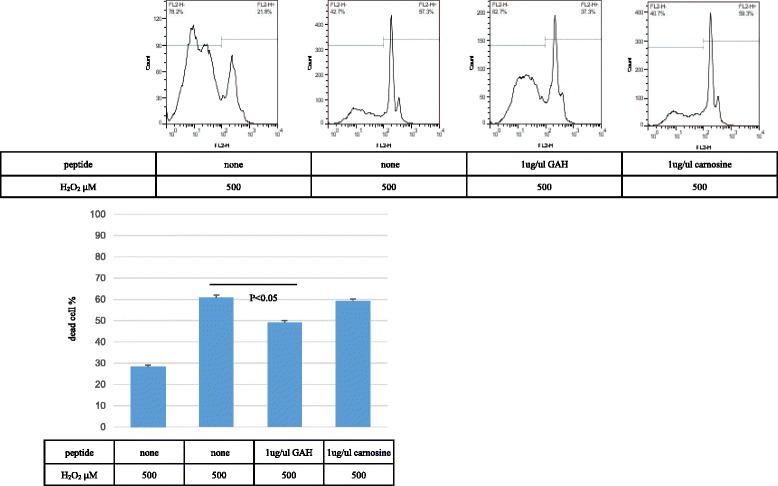
GAH protects PC12 cell death as determined by flow cytometric analyses. The cells were treated with 500 μM of hydrogen peroxide for 24 h, labeled with propidium iodide, and analyzed by flow cytometry. The upper image shows data from flow cytometric analyses. PI is shown as log fluorescence. The lower graphs show the percentage of dead cells in each experiment. All data are shown as mean ± SE of three independent experiments (*p* < 0.05)

Flow cytometric analyses were performed on the cells after propidium iodide (PI) staining to evaluate the effects of GAH on PC12 cells treated with 500 μM of hydrogen peroxide for 4 h. As shown in Fig. 6, the percentage of PI-positive PC12 cells was 28.13 ± 4.88% without hydrogen peroxide, and 60.95 ± 14.82% with 500 μM of hydrogen peroxide, after 4 h. GAH decreased the percentage of PI-positive PC12 cells treated with 500 μM hydrogen peroxide by 11.13% (from 60.95 ± 14.82% to 49.04 ± 10.49%; *p* = 0.0219). In contrast, carnosine did not significantly affect this parameter (reduced from 60.95 ± 14.82% to 59.16 ± 14.45%; *p* = 0.748). Thus, GAH exerted a cell protective effect superior to that of carnosine.

**Fig. 7:**
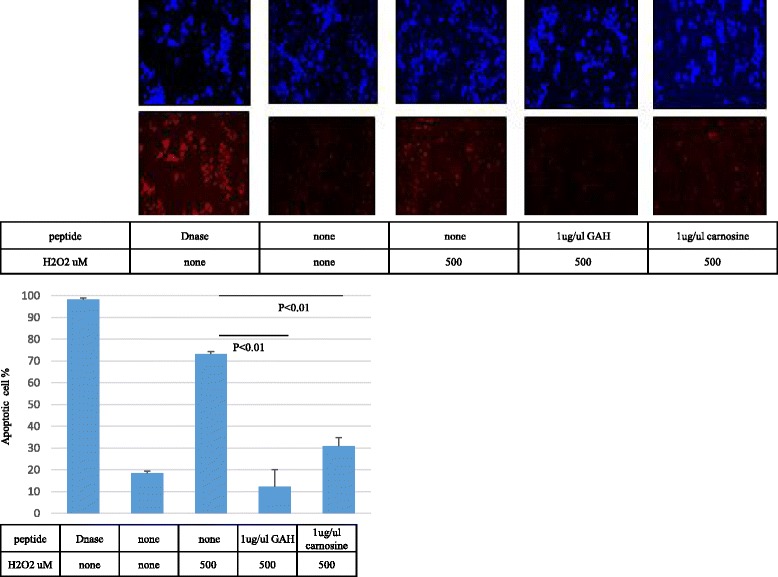
GAH decreases the number of TUNEL-positive PC12 cells. PC12 cells were exposed to 500 μM hydrogen peroxide for 24 h. The TUNEL assay was performed using the Click-iT TUNEL Alexa Fluor 594 Imaging Assay Kit. The TUNEL signal is shown in *red* (*top* of figure, *bottom* panels) and Hoechst 33,342 nuclear staining is shown in *blue* (*top* of figure, *top* panels). The *upper* panels are representative images of TUNEL staining. All data are shown as mean ± SE of three independent experiments (*p* < 0.01)

**Fig. 8:**
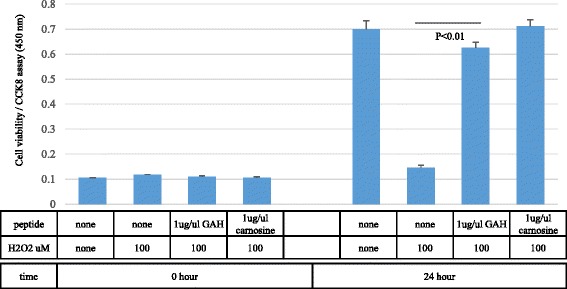
GAH protects PC12 cells as measured by the CCK-8 assay. PC12 cells were exposed to 100 μM hydrogen peroxide. Cell viability was measured using the CCK-8 assay at the start and after 24 h incubation. All data are shown as mean ± SE of three independent experiments (*p* < 0.01)

The percentage of TUNEL-positive PC12 cells was 18.4 ± 0.87% without hydrogen peroxide, and 73.1 ± 1.17% after treatment with 500 μM hydrogen peroxide for 24 h. GAH decreased the percentage of TUNEL-positive hydrogen peroxide-treated PC12 cells by 60.9% (from 73.1 ± 1.17% to 12.21 ± 0.078%; *p* < 0.01) (Fig. 7). Carnosine also decreased the percentage of TUNEL-positive PC12 cells (by 42%, from 73.1 ± 1.17% to 31 ± 3.75%; *p* < 0.01). GAH thus prevented apoptosis better than carnosine. Whether the viability of PC12 cells exposed to 100 μM hydrogen peroxide was protected by GAH was investigated using the CCK-8 assay. The results showed that cell viability (as measured at OD 450 nm) was significantly increased in the GAH (0.625 ± 0.023)- and carnosine (0.711 ± 0.026)-treated groups relative to controls (0.144 ± 0.012, p < 0.01) (Fig. 8). Thus, in this assay, there was no difference between GAH and carnosine.

**Fig. 9:**
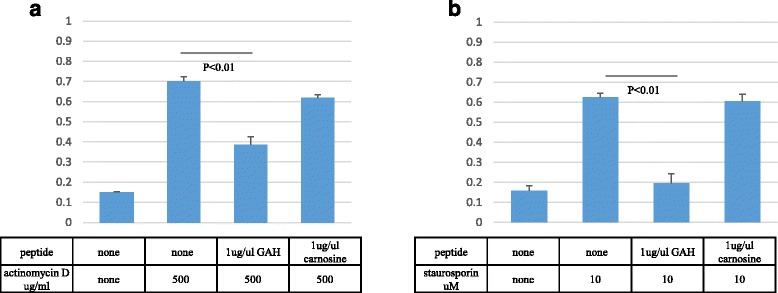
GAH protects against PC12 cell damage caused by actinomycin D and staurosporine. PC12 cells were exposed to 500 μg/mL of actinomycin D (**a**) or 10 μM of staurosporine (**b**) for 12 h. Cell viability was measured by the LDH leakage assay. All data are shown as mean ± SE of three independent experiments (*p* < 0.01)

We also examined the protective effects of GAH against actinomycin D and staurosporine-induced apoptotic cell death of PC12 cells [19, 20]. Cells were incubated with 500 μg/mL of actinomycin D or 10 μM of staurosporine for 12 h, and viability again measured by the LDH leakage assay. GAH did prevent cell death induced by actinomycin D (*p* < 0.001) and staurosporine (*p* < 0.001)) similar to its effects on hydrogen peroxide toxicity (Fig. 9).

**Fig. 10:**
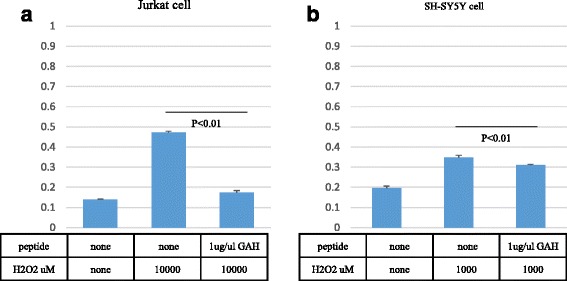
GAH protects against Jurkat and SH-SY5Y cell damage caused by hydrogen peroxide. Jurkat cells were exposed to 10,000 μM hydrogen peroxide for 4 h (**a**). SH-SY5Y cells were exposed to 1000 μM hydrogen peroxide for 24 h (**b**). Cell viability was measured by the LDH leakage assay. All data are shown as mean ± SE of three independent experiments (*p* < 0.01)

We also examined the effect of GAH on Jurkat cells (human lymphocyte cell type) and SH-SY5Y cells (human neuroblastoma cells) in culture. Jurkat cells were incubated with 10,000 μM of hydrogen peroxide for 4 h. GAH at 1 μg/μL decreased the amount of LDH release from Jurkat cells (*p* < 0.001) (Fig. 10a). We also incubated SH-SY5Y cells with 1000 μM of hydrogen peroxide for 24 h in the presence or absence of GAH. At 1 μg/μL, GAH also decreased the amount of LDH released from SH-SY5Y cells (*p* = 0.0039) (Fig. 10b). These findings indicate that GAH prevents Jurkat and SH-SY5Y cell membrane damage caused by hydrogen peroxide.

## Discussion

We screened newly synthesized histidine-containing tripeptides for their radical scavenging activity. GHH, AHH, AHA, AAH, AGH, GAH, AHG, HAG, HHH, GHA, and GGH were screened for their ability to decrease LDH leakage from PC12 cells treated with hydrogen peroxide. Of these peptides, GAH at 1 μg/μL had the strongest protective effect against cell damage as assessed by LDH leakage, by ethidium bromide staining, cell morphology, TUNEL assays, CCK-8 assays, and PI assays. GAH also protected Jurkat cells and SH-SY5Y cells; it may therefore have a protective effect against many different types of cells. GAH was not effective for SH-SY5Y cells compared to PC12 cells suggesting that effectiveness of GAH might depends on cell type.

Several histidine-containing dipeptides or tripeptides with antioxidant activity have been identified. Hartman et al. [21] have shown that carnosine is an efficient singlet-oxygen scavenger, quenching singlet oxygen more effectively than histidine. They also reported that carnosine, anserine, and histidine protect phages against gamma-irradiation, which gives rise to oxidative DNA damage. Tsuge et al. reported the isolation of a potent antioxidative peptide, Ala-His-Lys, from an egg white albumin hydrolysate [15]. Chen et al. reported that Pro-His-His was the most active antioxidant among the 28 synthetic peptides that were structurally related to Leu-Leu-Pro-His-His [16]. Saito et al. reported that Tyr-His-Tyr had a strong synergistic effect with phenolic antioxidants [17]. As reported here, GAH attenuated cell damage by hydrogen peroxide, suggesting that it might be an efficient singlet oxygen scavenger, similar to carnosine, Pro-His-His, and Tyr-His-Tyr.

GAH protected cells not only against hydrogen peroxide damage, but also prevented apoptosis induced by staurosporine and actinomycin D. Staurosporine is a broad-spectrum inhibitor of protein kinases, and has been widely used for the induction of apoptosis in diverse cellular models [22, 23]. Staurosporine preferentially activates the mitochondrial apoptotic pathway, relying on caspase activation to cause cell death. Actinomycin D, on the other hand, is a widely-used intercalating transcription inhibitor. [24]. Protection by GAH against hydrogen peroxide, staurosporine, and actinomycin D suggests that it might not only be a radical scavenger but could also protect by other mechanisms. The protective effects of carnosine include actions on glycolytic enzymes, metabolic regulatory activities, redox biology, protein glycation, glyoxalase activity, apoptosis, gene expression, and cancer cell metastasis [25].

In this study, we did not address the mechanism of how GAH attenuated cell death. Further studies will be needed to clarify the mechanism of GAH cytoprotection.

## Conclusions

The present studies showed that GAH has protective effect against cell damage determined by LDH leakage, by ethidium bromide staining, cell morphology, TUNEL assays, CCK-8 assays, and PI assays. GAH also protected Jurkat cells and SH-SY5Y cells. GAH might has a potential for cytoprotective agents.
